# Computational Analysis of AmpSeq Data for Targeted, High-Throughput Genotyping of Amplicons

**DOI:** 10.3389/fpls.2019.00599

**Published:** 2019-05-14

**Authors:** Jonathan Fresnedo-Ramírez, Shanshan Yang, Qi Sun, Avinash Karn, Bruce I. Reisch, Lance Cadle-Davidson

**Affiliations:** ^1^Department of Horticulture and Crop Science, The Ohio State University, Wooster, OH, United States; ^2^School of Integrative Plant Science, Cornell AgriTech, Geneva, NY, United States; ^3^Biotechnology Resource Center, Bioinformatics Facility, Institute of Biotechnology, Cornell University, Ithaca, NY, United States; ^4^United States Department of Agriculture – Agricultural Research Service, Grape Genetics Research Unit, Geneva, NY, United States; ^5^Shanshan Yang, Virginia G. Piper Center for Personalized Diagnostics, Biodesign Institute, Arizona State University, Tempe, AZ, United States

**Keywords:** molecular marker development, targeted amplicon sequencing, SNP markers, haplotype resolution, amplicon read counts, heterozygosity

## Abstract

Amplicon sequencing (AmpSeq) is a practical, intuitive strategy with a semi-automated computational pipeline for analysis of highly multiplexed PCR-derived sequences. This genotyping platform is particularly cost-effective when multiplexing 96 or more samples with a few amplicons up to thousands of amplicons. Amplicons can target from a single nucleotide to the upper limit of the sequencing platform. The flexibility of AmpSeq’s wet lab methods make it a tool of broad interest for diverse species, and AmpSeq excels in flexibility, high-throughput, low-cost, accuracy, and semi-automated analysis. Here we provide an open science framework procedure to output data out of an AmpSeq project, with an emphasis on the bioinformatics pipeline to generate SNPs, haplotypes and presence/absence variants in a set of diverse genotypes. Open-access tutorial datasets with actual data and a containerization open source software instance are provided to enable training in each of these genotyping applications. The pipelines presented here should be applicable to the analysis of various target-enriched (e.g., amplicon or sequence capture) Illumina sequence data.

## Introduction

A diverse array of genotyping platforms and molecular marker types have been utilized for various applications in plant species, including both nuclear and cytoplasmic loci, such as simple sequence repeats (SSR) and single-locus or multi-locus single nucleotide polymorphism (SNP) assays. SSRs are particularly well-suited for genetic mapping and comparative genomics because of their multi-allelic nature and high transferability among distinct species or genera (Arnold et al., 2002; Mnejja et al., 2010), which enables the analysis of complex diversity panels involving multiple species or interspecific hybridizations. However, SSR as a genotyping platform has its own disadvantages, being low-throughput, low-resolution, and labor-intensive (Deschamps et al., 2012). SNP microarrays emerged as an alternative high-throughput genotyping platform (Grattapaglia et al., 2011), however, regardless of their advantages, SNP microarrays are closed platforms, focused in commercially relevant species, suffering from ascertainment bias (Moragues et al., 2010), and resulting in poor flexibility and poor transferability across diverse germplasm (Thomson, 2014). In addition, the cost of microarray design is still a major obstacle in adopting SNP arrays (Miller et al., 2013; Myles et al., 2015), although efforts are being pursued to improve cost effectiveness (Chagné et al., 2019).

Next-generation sequencing (NGS) technology offers a potential opportunity for unbiased genotyping with high-throughput and low per-sample cost. Simultaneous marker discovery and genotyping delivers many benefits including availability of flanking DNA sequence information, high resolution, and high-sample throughput and scalability (multiplexed loci) (Elshire et al., 2011). However, for thorough characterization of specific loci, approaches such as genotyping-by-sequencing (GBS, Elshire et al., 2011) are not optimal. RAD-seq and GBS work on the premise of providing an overview of genome-wide polymorphisms, which is enhanced with the availability of pangenomes or haplotype maps (HapMaps, Bukowski et al., 2016) and full genome annotations. However, such resources may not exist for most taxa, and the use of such genome-wide polymorphism-discovery pipelines is focused only in discovering SNPs or Indels. In outcrossing plant species, information may be also limited, since an assumption under which these pipelines were originally conceived is that all the polymorphisms will be in homozygous state, enabling low sequencing depth aided by posterior imputation and augmentation. When the characterization of heterozygous loci is needed, deeper sequencing is required to effectively differentiate the zygosity. From a technical perspective, missing data, genotyping errors, and heterozygote under-calling are common in genome-wide studies using high-multiplexing coupled with NGS, which results in uneven sequencing depth across sites (Beissinger et al., 2013; Bianco et al., 2014; Glaubitz et al., 2014; Swarts et al., 2014). From a biological point of view, lack of knowledge regarding rapid linkage disequilibrium (LD) decay, lack of structured or inbred germplasm, and large-scale genome structure variation coupled with lack of haplotype information makes it impractical to do genotype imputation in highly diverse plant species. From a practical perspective, long turn-around time from sample collection to data analysis, computational challenges in hardware needed for computation, data processing and result interpretation may be main obstacles for some researchers and small research groups.

The AmpSeq genotyping platform (Yang et al., 2016) allows high-throughput and highly multiplexed genotyping of known and unknown polymorphisms, which may or may not be associated to a trait. Initially developed to target SNPs (or short sequence targets around 45 bp), AmpSeq has been expanded to target amplicons of up 600 bp. This strategy exploits the high-throughput data yield and massive multiplexing of Illumina technology, which results in low-cost genotyping. The strategy is based on having a target region or sequence for which primer design is possible [e.g., Quantitative Trait Loci (QTL), Single Sequence Repeats (SSRs) or cloned genes]. Subsequently, primers are designed flanking SNP markers or longer sequence targets, and then deployed to the Illumina sequencing technology after polymerase chain reaction (PCR) and multiplexing. Finally, the data yielded from the sequencer is processed through bioinformatics pipelines for SNP calling and read count matrices.

**FIGURE 1:**
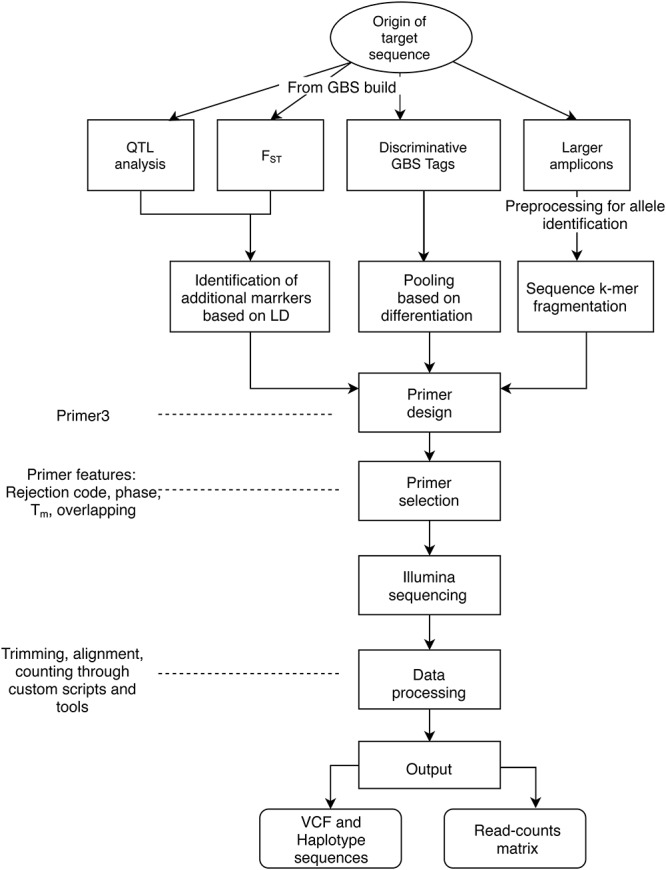
General workflow of the AmpSeq strategy. Here, we describe three sources of short AmpSeq markers derived from GBS 64 bp sequences (QTL analysis, F_ST_, Discriminative tags) as well as longer amplicons from SSRs or gene sequences, and considerations for primer design and selection. A primary focus of the documentation provided here is on data processing and interpretation.

The objectives of applying AmpSeq may be to develop markers for high-throughput genotyping or to explore the sequence diversity of the targets, and then use the output data for distinct purposes, such as population genetics or allele discovery and mining. The target sequences have been primarily based on four sources (Figure 1): (a) QTL regions from analyses of segregating families, (b) target signatures of germplasm differentiation identified through fixation index (F_ST_), (c) GBS tags (64 bp sequences) identified through discriminative pooled-sampling of germplasm, and (d) already known gene sequences processed through amplicon sequencing data for larger target regions (>200 bp) (Fresnedo-Ramírez et al., 2017), for which genotyping is more expensive.

In this document, a pipeline for the processing of AmpSeq data prior to interpretation is presented. Emphasis will be given to three main types of data: SNPs, haplotypes and amplicon read counts. The basic information is presented here, along with key output files and their descriptions. Data and a more comprehensive step-by-step procedure and well as a containerization instance with the software required are available through the GitHub and Singularity Hub repositories.

Note that several variants of amplicon sequencing are available through commercial providers. In addition, sequence capture approaches have a parallel concept of target enrichment prior to sequencing and are available through an array of commercial providers. Whether genotyping in-house or with a service provider, in most cases these diverse target-enriched Illumina sequence data types could be analyzed with the pipelines described here, particularly if reference sequences (for SNP calling), 5^′^ sequence of the target region (for haplotyping), or a larger component of target sequence (for read counts) is available.

## Materials

### Hardware

The requirements of hardware to analyze AmpSeq data will depend mostly on the number of samples to be analyzed, the type of data to be generated, and the sequencing platform used for the generation of the amplicon sequences. Up to 2500 samples have been successfully analyzed using the procedure described herein by using a workstation with 32 GB of RAM and a quad-core microprocessor. In terms of storage, for those 2500 samples processed with a mixture of small (up to 50 bp) and medium (up to 200 bp) length amplicons, less than 100 GB were required. The final outputs of the AmpSeq computational analysis are small, usually VCF files or text files of KB or MB (in the example provided here, the basic data is ∼300 MB, and after finishing all the routines the directory is ∼6 GB).

If the number of samples is greater than 3000 and hundreds of amplicons of distinct lengths are going to be analyzed, it is recommendable to use dedicated workstations or high-performance computing clusters, in order to accelerate the processing and also obtain support related to customizations of the scripts and container provided in this communication. In addition, having access to institutional or dedicated computing clusters allows both new and experienced users to save time in dealing with new installations and configuring access to software.

### Software

The computational analysis of AmpSeq data requires the use of the command line and the installation of Perl 5.16 and above, Java 8 and above. The software packages (and their dependencies) considered for this pipeline include: BWA (Li and Durbin, 2009, 2010; Li, 2013), samtools (Li et al., 2009), Trimmomatic (Bolger et al., 2014), GATK (McKenna et al., 2010; Van der et al., 2013), BLAST (Camacho et al., 2009), FLASh (Magoč and Salzberg, 2011), clustalw (Larkin et al., 2007), clustal-omega (Sievers et al., 2011), jellyfish (Marçais and Kingsford, 2011), R Statsitical Language and Framework (R Core Development Team, 2017), and vcftools (Danecek et al., 2011), in their most recent stable releases as of February 2019.

### Files and Scripts

Supporting this manuscript, a compilation of examples of input files and scripts is available at the AmpSeq repository: https://github.com/JFresnedo/AmpSeq. Also, a Singularity Container with all the software needed to run this pipeline is available through https://singularity-hub.org/collections/2392. Thus, as an exercise of open science, users may use the pipeline, get familiar with it and replicate the results shown here. This pipeline is composed of four routines: Primer Design, SNP calling, Haplotyping, and Read Counts (Figure 1). The scripts are written in Perl. Each section of the routine at GitHub is a directory that includes scripts and sample files. Most files are text based. Below, a succinct description of each routine is provided along with the direct link to the subdirectory in the AmpSeq repository to provide access to data, additional instruction and scripts.

#### Primer Design

These procedures design target-specific primers to use for library preparation. This routine was developed with the purpose of designing primers to amplify GBS-tags containing SNPs in a given chromosomic region of interest (e.g., a QTL). This routine requires a SAM file from the SNP discovery pipeline (in this case Tassel GBS pipeline v. 3), a VCF with the polymorphisms discovered out of the SNP-discovery pipeline, and a fasta file with the reference sequence of the chromosome, contig or scaffold. A subsample of the GBS data analyzed in Yang et al. (2016) is provided for the analysis of *REN2* powdery mildew resistance in the segregating population “Horizon” × Illinois 547-1. Example data are available at: https://github.com/JFresnedo/AmpSeq/tree/master/1_Primer_Design.

#### SNP Calling

These are procedures to discover SNPs relative to a reference sequence. This routine automatically trims sequence reads for adaptors, length and quality to subsequently process reads through procedures of aligning against reference, sorting from Picard and calling SNPs through the HaplotypeCaller routine of GATK. The outcome is a VCF file with the list of SNPs per sample analyzed. The data for this section and Haplotyping consist of paired-ended sequencing data of two parents (Parent1 and 2) and 10 progenies (Progeny01 to 10) for which amplicons targeted the powdery mildew resistance loci *RUN1* (chr12), *REN1* (chr13), *REN6* (chr9), and *REN7* (chr19) and the downy mildew resistance locus *RPV12* (chr14). Example data are available at: https://github.com/JFresnedo/AmpSeq/tree/master/2_SNP_calling/

#### Haplotyping

PCR amplicons can contain one or more polymorphic sites that together can be considered a unique haplotype, or unique sequence read. The haplotyping procedure identifies unique haplotypes as alleles and associates allelic combinations with each sample. This routine automatically trims the sequencing data (for adaptors, length, and quality) to subsequently process unique reads through multiple sequence alignment using Clustal Omega and sorting from Picard. If reference sequences are provided for the amplicons analyzed, it is possible also to call SNPs by using the HaplotypeCaller routine of GATK. The expected outcome is Variant Call Format (VCF)-type file and a list of haplotype sequences. The data for this section and SNP Calling consist of two parents (Parent1 and 2) and 10 progenies (Progeny01 to 10) described above. Example data are available at: https://github.com/JFresnedo/AmpSeq/tree/master/3_Haplotyping.

#### Read Counts

This procedure quantifies reads of known sequences. For this rapid routine written in a perl script, the reads are parsed using the forward primers or sequences of the amplicons and enumerated. This procedure is useful when presence/absence markers or a specific allele are used to assay the sample population. The expected outcome is a text file containing a matrix of read counts. A subsample of the data for amplicons generated through single-ended sequencing targeting the powdery mildew resistance loci *RUN1* and *REN4* is provided from Fresnedo-Ramírez et al., 2017. Example data are available at: https://github.com/JFresnedo/AmpSeq/tree/master/4_Read_Counts.

## Approaches and Results

To get started with AmpSeq, the use of existing primers that are known to produce amplicons in any platform is the simplest way. Nearly all of these (>95%, in our experience) will return data in AmpSeq multiplexes. Similarly, simply using Primer3 (Koressaar and Remm, 2007; Untergasser et al., 2012) to design new primers to any sequence of interest is simple and most (70%, in our experience) will return data in AmpSeq multiplexes. Higher rates are obtained when the sequence diversity of the target samples is included in primer design, to ensure primer binding on conserved sites. Below, a more complex approach of primer design from GBS data is presented.

### Primer Design Based on Genotyping-by-Sequencing (GBS) Data

As described by Yang et al. (2016), one can design primers for posterior SNP calling using information yielded through the TASSEL-GBS pipeline (Glaubitz et al., 2014) and QTL mapping results, tests of fixation index (F_ST_), or other strategies for marker-trait association. This procedure begins by defining the most significant marker (anchor marker) and two flanking markers (defining the confidence interval) in a QTL region. In the case of data from F_ST_, the target region should be already known, then, what is needed is an interval supported by F_ST_ > 0.35, which will suggest deviations in the allele frequencies with respect to the germplasm pools considered, and therefore a target region of differentiation (Hey and Pinho, 2012; Porto-Neto et al., 2013). Markers and primers yielded from this procedure is provided in the Supplementary File S1.

### SNP Calling Using GATK

The Perl wrapper script run_gatk2.pl trims sequence reads to 45 bp before aligning them to the reference genome. It produces one SAM file per individual, converted to BAM files for sorting, cleaning and merging before using GATK for SNP calling and VCF file generation. That VCF file contains all the AmpSeq SNPs identified in the analysis and can be manipulated using vcftools or TASSEL (Bradbury et al., 2007) for downstream analyses. Markers and genotypes in the distinct chromosomes (based on the VCF file) is provided in the Supplementary File S2.

### Haplotyping for the Development of AmpSeq for Markers

The amplicon sequencing technology is flexible with respect to the type of designs that one can develop. The initial emphasis was on short, 45 bp markers converted from GBS data to genotype SNPs and presence/absence sequences, or tags. In addition, longer amplicons from gene regions, SSRs, or other sources can be genotyped, taking advantage of pair-ended sequence technology. This enables cost-effective integration of diverse marker platforms, for applications such as marker-assisted selection. When multiplexing amplicons of different lengths, shorter amplicons tend to return significantly more data than longer amplicons. Further, data analysis is simplified when the range of amplicon sizes is restricted.

In this example, data analysis focuses on longer amplicons, specifically SSR markers (Table 1). These primers come from fluorescently labeled PCR products analyzed via capillary electrophoresis, an approach that is reliable in the prediction of the resistance but with a limited capacity of multiplexing.

**Table 1:** Output of the haplotype alleles for two parent samples and seven markers in the data provided at https://github.com/JFresnedo/AmpSeq/tree/master/3_Haplotyping.

		Sample	
Marker	Haplotypes (frequency)^a^	Parent1^b^	Parent2
Ren1_SC47_6	1(0.38);3(0.29);2(0.25);4(0.08);	2/3:1587,1511	1/4:2927,2653
Ren6_PN9_063	2(0.29);4(0.29);3(0.12);1(0.12);10(0.08);11(0.04);9(0.04);	9/10:7,4	1/3:610,170
Ren6_PN9_068	4(0.32);2(0.27);3(0.27);1(0.14);	3/4:8,3	1/2:35,14
Ren7_PN19_018	1(0.50);2(0.33);4(0.08);3(0.08);	2/4:141,130	1/1:1752
Ren7_VVin74	1(0.33);4(0.29);2(0.25);3(0.12);	2/4:139,42	3/1:175,117
Rpv12_UDV014	4(0.32);2(0.18);1(0.18);5(0.18);6(0.14);	1/2:76,63	./.:0
Rpv12_UDV370	1(0.33);2(0.33);3(0.17);5(0.12);4(0.04);	3/1:3735,3018	5/2:4379,3709


*^a^Each haplotype allele, or unique sequence, is assigned a unique number (here, 1–11). The sequence corresponding to the allele number can be found in the haplotype2fasta folder. In parentheses, this column presents each allele’s frequency across all samples. ^b^The haplotype data for each sample is represented as allele1/allele2:reads1,reads2. Missing data are denoted as./.:0, and homozygous sites have a repeated allele number with a single read count (e.g., 1/1:1752).*

### Read Counts Matrix

GBS tags are reference-independent sequences unbiased by the genomic background of samples and are particularly useful for introgression of traits from wild accessions lacking a reference genome. Marker design uses the tags-by-taxa (TBT) file generated during the TASSEL-GBS pipeline, prior to read alignment to a reference, to detect differential presence/absence of tags with respect to phenotypic data (such as presence/absence of disease resistance). Tag sequences differentially enriched in a given germplasm pool can be used for BLAST query to identify redundant tags (optional) and Primer3-based primer design for the development of 45–50 bp presence/absence AmpSeq markers.

After sequencing, the output of tag_presence.pl is a matrix with the counts for every tag (rows) in every individual (columns), with normalized and non-normalized versions. The normalized version shows values normalized by total reads then multiplied times 1,000,000, which can be useful to deal with uneven DNA concentration and sequencing depth (Table 2).

**Table 2:** Output of the read counts in matrix_normalized.txt for four samples and seven markers in the data provided at https://github.com/JFresnedo/AmpSeq/tree/master/4_Read_Counts.

	Sample			
Marker	7_18_06_3	7_18_06_5	7_20_03_1	7_20_03_3
Ren4_Tag4	0	22	9211	11,013
Ren4_Tag6	0	0	2414	3607
Ren4_Tag9	118	156	99	143
Ren4_Tag16	0	0	172	239
Ren4_Tag18	0	0	74	24
Ren4_Tag19	0	0	99	96
Run1_CB3334_Haplo64	0	0	0	0


**FIGURE 2:**
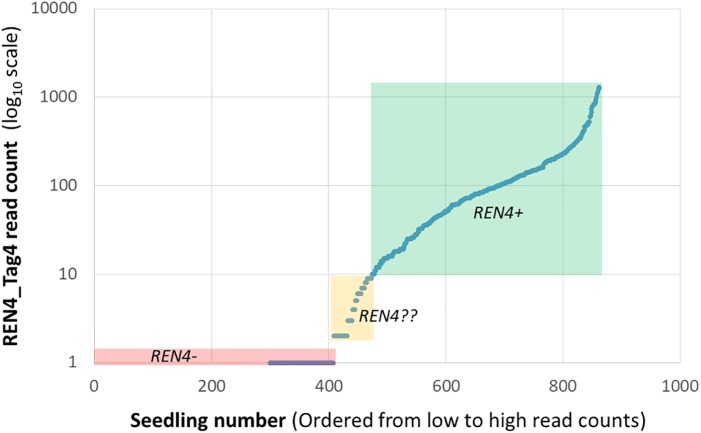
Distribution of read counts for the REN4_Tag4 sequence for a marker-assisted selection pseudotestcross from Fresnedo-Ramírez et al. (2017), illustrating continuously variable read counts for this presence/absence marker. Three distributions based on arbitrary thresholds are highlighted here: 408 samples with 0 or 1 read that are likely REN4- in a red box, 389 samples with 10 or more reads that are likely REN4+ in a green box, and 65 samples with 2–9 reads with uncertain REN4?? status in a yellow box.

The files can be easily opened in Excel, where it is possible transpose the matrix and start to manipulate the information, such as by adding phenotypic information. The subsequent manipulation of the information depends on the data quantity and application. Often, quantitative read counts are continuously variable even for alleles that are present or absent, and mean read depth per marker is not even across loci. For these reasons, read count standardization and thresholding can be helpful (Fresnedo-Ramírez et al., 2017). One initial strategy is to plot the values of read counts on a logarithmic scale versus samples, sorted from lowest to highest read count. While that is not useful for the small data sets provided here, for a marker-assisted selection pseudotestcross where half of the seedlings should be positive for *REN4* based on Mendelian genetics, 10 read counts was selected as the threshold to call for the presence of Tag4, given the discontinuity of the line at very low values (Figure 2). The results fit with expectations, as there were 408 seedlings with 0 or 1 read counts and 389 seedlings with 10 or more reads. The 65 seedlings with 2–9 reads considered uncertain could be discarded as REN4- (negative for the gene) or kept and re-genotyped.

Establishing a threshold may be experiment specific, in part because sequencing depth is dependent on sequencing platform and the number and identity of multiplexed markers or samples, which may change from one batch to the next. For example, note that the maximum read count in Figure 2 was 10-fold lower than those for 7_20_03 samples in Table 2. Therefore, one must be cautious and carefully consider the experimental design and analysis: genotype standard DNA controls from well-known vines having the locus of interest or not, including duplicated samples, and analyze read count distribution in wells known to lack the locus. When more rigor is needed or with larger data sets, routines for ordinal (for disease resistance or compound concentration) or nominal (for categories that do not represent a gradient) logistic regression are useful. While normality of the read count residuals is not assumed in logistic regression, normalization of the data may alleviate some issues from uneven sequencing, like outlier individuals with very high values.

The read counts are somewhat influenced by the zygosity of the locus, even though the high number of AmpSeq PCR cycles makes the read counts quantitatively imprecise. Thus, loci that are in a homozygous state usually have higher read counts in comparison with those in heterozygous states (not necessarily double). Sometimes such a distinction may be useful to discriminate individuals resulting from unintended self-fertilization. Having selfs in the dataset may significantly deviate the thresholds to call for the presence/absence of the marker; therefore, it is highly desirable to analyze for possible selfs beforehand, which we typically do using data from biallelic SNP markers with known parental alleles.

## Conclusion

AmpSeq provides flexibility to address the limitations of other marker platforms, particularly in high diversity, heterozygous taxa like *Vitis*, where wide crosses are used for trait introgression. Here we describe the computational tools we developed to integrate short and long amplicon markers containing one or several polymorphisms or varying by presence/absence. Much of our work has focused on conversion either of GBS SNPs or tags to short amplicons, or of SSRs or gene sequences to long amplicons. Our applications have included marker assisted selection, gene profiling, population genetics, and detection of gene edits. In these efforts, grapevine has provided a model to adapt AmpSeq technology to perennial heterozygous crops, which face distinct challenges compared to inbred model crop species. Now, AmpSeq is being applied beyond crops to fungal pathogens and non-culturable organisms for which DNA quantity or quality limits other genotyping approaches. Given this flexibility, we anticipate the tools and resources provided here could be widely used for diverse organisms and applications.

## Author Contributions

AK, JF-R, SY, LC-D, and QS analyzed the data. BR developed the germplasm. LC-D, QS, and JF-R planned the study. AK, JF-R, SY, and LC-D wrote the manuscript. All authors read and approved the final manuscript.

## Disclaimer

Mention of trade names or commercial products is solely for the purpose of providing specific information and does not imply recommendation or endorsement by the U.S. Department of Agriculture. USDA is an equal opportunity provider and employer.

## Conflict of Interest Statement

The authors declare that the research was conducted in the absence of any commercial or financial relationships that could be construed as a potential conflict of interest.

## Supplementary Material

FILE S1 Output of primer design from genotyping-by-sequencing (GBS) markers linked with *REN2* powdery mildew resistance in the segregating population “Horizon” × Illinois 547-1, corresponding to the data provided at https://github.com/JFresnedo/AmpSeq/tree/master/1_Primer_Design. Definitions of the column headers are provided in the second tab of Supplementary File S1.

FILE S2 Output of markers in the relevant chromosomes from paired-ended sequencing data of two parents (Parent1 and 2) and 10 progenies (Progeny01 to 10) for which amplicons targeted the powdery mildew resistance loci *RUN1* (chr12), *REN1* (chr13), *REN6* (chr9), and *REN7* (chr19) and the downy mildew resistance locus *RPV12* (chr14). These data are available at: https://github.com/JFresnedo/AmpSeq/tree/master/2_SNP_calling/.
